# Phorbol ester-induced angiogenesis of endothelial progenitor cells: The role of NADPH oxidase-mediated, redox-related matrix metalloproteinase pathways

**DOI:** 10.1371/journal.pone.0209426

**Published:** 2019-01-15

**Authors:** Tao-Cheng Wu, Chia-Chi Chang, Hsin-Bang Leu, Po-Hsun Huang, Shing-Jong Lin, Jaw-Wen Chen

**Affiliations:** 1 Division of Cardiology, Department of Medicine, Taipei Veterans General Hospital, Taipei, Taiwan, ROC; 2 Cardiovascular Research Center, National Yang-Ming University, Taipei, Taiwan, ROC; 3 Institute of Pharmacology, National Yang-Ming University, Taipei, Taiwan, ROC; 4 Healthcare and Management Center, Taipei Veterans General Hospital, Taipei, Taiwan, ROC; 5 Department of Medical Research, Taipei Veterans General Hospital, Taipei, Taiwan, ROC; University of Edinburgh, UNITED KINGDOM

## Abstract

Endothelial progenitor cells (EPCs) may contribute to ischemia-induced angiogenesis in atherosclerotic diseases. The protein kinase C (PKC) family is involved in the regulation of angiogenesis, however the role of PKCα in EPCs during angiogenesis is unclear. The aim of this study was to evaluate the role of PKCα in EPCs during angiogenesis. Phorbol-12-myristate-13-acetate (PMA), a PKCα activator, significantly increased the activity and expression of matrix metalloproteinases (MMP) -2 and -9 in human (late outgrowth) EPCs in vitro. The MMPs promoted the migratory function and vascular formation of EPCs, which then contributed to neovascularization in a mouse hindlimb-ischemia model. Reactive oxygen species derived from nicotinamide adenine dinucleotide phosphate (NADPH) oxidase enhanced the expression of MMPs to increase the bioactivity of EPCs during angiogenesis. The mitogen-activated protein kinase (MAPK) signal pathway was associated with the activation of NADPH oxidase. PMA extensively activated the extracellular signal–regulated kinase (Erk) signal pathway to increase the expression of MMP-9. PMA also activated the p38, Erk, and c-Jun N-terminal kinase signal pathways to increase the expression of MMP-2. PMA-stimulated EPCs enhanced neovascularization in a mouse model of hindlimb ischemia via nuclear factor-κB translocation to up-regulation of the expression of MMP-2 and MMP-9. PMA could activate PKCα and promote the angiogenesis capacity of human EPCs via NADPH oxidase-mediated, redox-related, MMP-2 and MMP-9 pathways. The PKCα-activated, NADPH oxidase-mediated, redox-related MMP pathways could contribute to the function of human EPCs for ischemia-induced neovascularization, which may provide novel insights into the potential modification of EPCs for therapeutic angiogenesis.

## Introduction

Angiogenesis plays a crucial role in tissue repair after ischemia that occurs in coronary artery disease, diabetes mellitus, stroke, and peripheral artery diseases. Various types of cells are involved in angiogenesis, including endothelial cells, monocytes, endothelial progenitor cells (EPCs), and others. EPCs are bone marrow–derived cells with the ability to differentiate into endothelial-like cells (so-called late outgrowth EPCs) and to regenerate endothelial cells [1, 2]. The migration, proliferation, and capillary tube formation of EPCs lead to neovascularization [3].

Hypoxia can induce angiogenesis through hypoxia inducible factor-1 (HIF-1) and vascular endothelial growth factor (VEGF)[4]. VEGF is an angiogenic factor that triggers angiogenesis via the extracellular signal–regulated kinase (Erk), protein kinase B (Akt), and endothelial nitric oxide synthase (eNOS) signal pathways [5]. In addition to VEGF, matrix metalloproteinases (MMPs) are also involved in angiogenesis and wound healing and can degrade extracellular matrix proteins, contributing to cell migration, proliferation, and differentiation [6]. MMP-2 and MMP-9 have been associated with the proliferation and migration of EPCs in ischemia-induced angiogenesis [7]. In addition, MMP-9 has also been reported to increase the expression of VEGF to promote angiogenesis [8].

Protein kinase C (PKC), a family of serine/threonine kinases, is divided into three groups, including conventional PKC (α, γ, and β), novel PKC (δ, ε, θ), and atypical PKC (η). Different PKC isoforms may have specific and opposing functions in vascular formation. For example, PKCδ has been shown to inhibit the differentiation of epididymal fat endothelial cells in rats [9]. PKCα has been shown to promote the angiogenic activity of human endothelial cells via the induction of VEGF [10]. In addition, the down-regulation of PKCα in human umbilical vein endothelial cells has been shown to inhibit vascular formation [11]. PKC has also been shown to be involved in the expression of platelet-derived growth factor C in hyperglycemic endothelial cells and may be related to angiogenesis in patients with diabetes [12]. However, little is known about the mechanistic role of PKCα activation in EPCs with regards to angiogenesis.

In the present study, we tested the hypothesis that activation of PKCα-related redox-sensitive pathways may improve EPC function for angiogenesis, phorbol-12-myristate-13-acetate (PMA), a well-known PKC activator, was used to evaluate the effects of PKCα activation on the angiogenic capacity of human (late outgrowth) EPCs, which were then transplanted to facilitate neovascularization in a mouse model of hindlimb ischemia. The mechanisms of PKCα activated, redox-related MMPs pathways were also investigated with regards to the angiogenesis capacity of EPCs. Our findings may provide novel insights into the potential modification of human EPCs for in vivo angiogenesis.

## Materials and methods

The Taipei Veterans General Hospital’s Institutional Review Board for Research approved the study protocol (VGHTPE IRB # 2011-09-015IC). All of the participants provided written informed consent. They were recruited through relevant information. All animals were housed and handled in accordance with criteria outlined in the National Institutes of Health ‘‘Guide for Care and Use of Laboratory Animals”. The study protocol was approved by the Institutional Animal Care and Use Committee (IACUC) of Taipei Veterans General Hospital, Taipei Taiwan. (Approval number: IACUC_2013–056). The animals were raised according to the regulations of the Animal Care Committee of National Yang-Ming University and the IACUC of Taipei Veterans General Hospital. All efforts were made to ameliorate animal suffering. The animals were sacrificed by the injection of sodium phenobarbital.

Phorbol-12-myristate-13-acetate (PMA), a PKCα activator, was purchased from Sigma (St. Louis, MO, USA). Culture media were purchased from Lonza (Walkersville, MD, USA). PD98059 (Erk inhibitor), SP600125 (JNK inhibitor), SB203580 (p38 inhibitor), Gö6976 (PKCα inhibitor), DPI (NADPH oxidase inhibitor), LY294002 (PI3-kinase inhibitor), and PDTC (a nuclear factor κB [NF-κB] inhibitor) were purchased from Enzo Life Science (New York, USA). Edaravone (free-radical scavenger) was purchased from TOCRIS (Britol, United Kingdom). Mouse monoclonal antibodies against phospho-p44/42 MAPK, phospho-SAPK/JNK, and rabbit polyclonal antibodies against p44/42 MAPK, SAPK/JNK, phospho-p38 MAPK, p38 MAPK, and MMP-2 were purchased from Cell Signaling Technology (Danvers, USA). Mouse monoclonal antibody against MMP-9 was purchased from Calbiochem (San Diego, CA, USA). Mouse monoclonal antibodies against phospho-Akt, Akt, p47phox, p65, and rat monoclonal antibody against anti–mouse CD31 were purchased from BD Pharmingen (New Jersey, USA). The fluorescein isothiocyanate (FITC)–conjugated Affinipure donkey anti-rat IgG (H+L) antibody was purchased from Jackson ImmunoResearch Laboratory (West Grove, PA, USA). The p47 small interfering RNA (siRNA), MMP-2 siRNA, and control siRNA (scrambled negative control containing random DNA sequences) were purchased from Santa Cruz Biotechnology (Santa Cruz, CA, USA). MMP-9 siRNA was purchased from Invitrogen (Carlsbad, CA, USA). DAPI solution was purchased from Millipore (Billerica, MA, USA). Dil-Ac-LDL was purchased from Biomedical Technologies (Stoughton, MA, USA). All other chemicals of reagent grade were obtained from Sigma.

### Isolation and cultivation of human EPCs

Total mononuclear cells (MNCs) were isolated from peripheral blood collected from healthy young human volunteers (who were recruited through relevant information) using density gradient centrifugation with Histopaque-1077 (1.077 g/mL; Sigma). Briefly, MNCs (5 × 10^6^) were plated in 2 mL of endothelial growth medium (EGM-2 Bullet Kit System; Lonza), with supplements (hydrocortisone, R3-insulin-like growth factor 1, human endothelial growth factor, VEGF, human fibroblast growth factor, gentamicin, amphotericin B, vitamin C, and 20% fetal bovine serum) on fibronectin-coated 6-well plates. Culture medium was replaced every 4 days. Colonies of (late outgrowth) EPCs appeared between 2 and 4 weeks. The (late outgrowth) EPCs exhibited a “cobblestone” morphology and a monolayer growth pattern typical of mature endothelial cells at confluence [13].

### Cell viability assay

The cell viability of late EPCs was determined by 3-(4,5-dimethylthiazol-2-yl)- -2,5,diphenyltetrazolium bromide (MTT) assay, respectively. The late EPCs were supplemented with MTT (0.5 mg/ml; Sigma) and incubated for 4 h for the proliferation assay. Blue formazan was dissolved with dimethyl sulfoxide and measured at 550/650 nm.

### Transplantation of cells in the mouse model of ischemia-induced angiogenesis

Six-week-old male NU/NU mice (n = 6) were purchased from the National Laboratory Animal Center (Taipei, Taiwan). The animals were raised according to the regulations of the Animal Care Committee of National Yang-Ming University. Unilateral hindlimb ischemia was induced in the second week by excision of the right femoral artery. Briefly, the proximal and distal portions of the right femoral artery and the distal portion of the right saphenous artery were ligated. The arteries and all side branches were then dissected free and excised. Hindlimb blood perfusion was measured with a laser Doppler perfusion imaging system (Moor Instruments Limited, Devon, UK) before and after the surgery, and weekly thereafter. The results were expressed as the ratio of the perfusion in the ischemic versus that in the non-ischemic limb. A solution of 1 × 10^6^ DiI-labeled cells in phosphate-buffered saline (PBS) was then injected into the ischemic hindlimb area after surgery (n = 6).

### Quantification of cell implantation in ischemic hindlimbs

DiI-labeled cells were injected into the ischemic hindlimbs of nude mice. After 4 weeks, the ischemic hindlimbs were harvested, and tissue sections were embedded and sectioned. Five fields from four tissue sections were randomly selected, and the number of DiI-labeled cells was counted in each field.

### Histologic analysis

Adductor muscles were incubated in a 30% sucrose solution for 24 h, embedded in OCT Compound (Sakura Finetek, Tokyo, Japan), and frozen in liquid nitrogen. For capillary density measurement, two sections taken approximately 3 mm apart were used. The sections, were fixed with methanol for 10 min and then washed briefly with PBS, before being stained with a monoclonal rat anti-murine platelet–endothelial cell adhesion molecule-1 (CD31) antibody (1:200; BD Pharmingen) at 37°C for 2 h, followed by incubation with an FITC-conjugated donkey anti-rat antibody (Jackson ImmunoReasearch). Capillaries were counted in three cross sections and analyzed for each animal; then ten different fields from each tissue preparation were randomly selected (n = 6 in each group). The results were calculated as capillaries per myocyte.

### Western blot analysis

After incubation, the cells were washed, scraped, collected, and centrifuged at 12,000 × *g* at 4°C for 1 h to yield the whole-cell extract. Samples were denatured, subjected to sodium dodecyl sulfate–polyacrylamide gel electrophoresis (SDS-PAGE), and transferred to a polyvinylidene difluoride membrane. The membranes were incubated with an anti-MMP-9, anti-MMP-2, anti-p65, anti-p-Erk, or anti-p47 antibodies for 24 h, followed by incubation with anti-rabbit or anti-mouse antibodies for 1 h. The immunoreactive bands detected by enhanced chemiluminescence reagents were developed using Hyperfilm-ECL (Amersham GE Healthcare, Chicago, USA).

### Transient transfection with small interfering RNA

SMARTpool RNA duplexes corresponding to human MMP9, MMP-2, and p47phox, and control small interfering RNA [(si)RNA] were obtained from Santa Cruz Biotechnology. Transient transfection of siRNAs was performed using Oligofectamine transfection reagent. siRNA (100 nmol/L) was formulated with oligofectamine transfection reagent according to the manufacturer’s instructions.

### Gelatin zymography

MMP-9 and MMP-2 activities in late cultured EPCs were determined using SDS-PAGE gelatin zymographic analysis. The samples were diluted in sample buffer (2% SDS, 125 mM Tris-HCl, pH 6.8, 10% glycerol, and 0.001% bromophenol blue) and subjected to electrophoresis on 10% SDS-PAGE co-polymerized with gelatin (1%) as the substrate. After electrophoresis, the gel was incubated for 1 h at room temperature in a 2% Triton X-100 solution, washed two times with water, and incubated at 37°C for 36 h in Tris-HCl buffer, pH 7.4, containing 10 mM CaCl_2_. The gel was stained with 0.05% Coomassie Brilliant Blue R-250, and then destained with 30% methanol and 10% acetic acid. Gelatinolytic activities were detected as unstained bands against the background of Coomassie Blue–stained gelatin. MMP-2 and MMP-9 were identified as bands at 72 and 92 kDa, respectively.

### EPC migration test

The migration of late EPCs was evaluated by a modified Boyden chamber assay (Transwell, Coster; Sigma-Aldrich, USA). Briefly, isolated EPCs were detached as described above with trypsin/EDTA, and then 4 × 10^4^ late EPCs were placed in the upper chamber of 24-well Transwell plates with polycarbonate membrane (8-μm pores) with serum-free endothelial growth medium; VEGF (50 ng/mL) in medium was placed in the lower chamber. After incubation for 24 h, the membrane was washed with PBS and fixed with 4% paraformaldehyde. The membrane was then stained using hematoxylin solution and was carefully removed. The magnitude of the migration of the late EPCs was evaluated by counting the migrated cells in six random high-power (100 ×) microscopic fields.

### EPC tube formation assay

An in vitro tube-formation assay was performed with an In Vitro Angiogenesis Assay Kit (Chemicon, Tamecula, USA). ECMatrix gel solution was thawed at 4°C, mixed with ECMatrix diluent buffer, and placed in a 96-well plate at 37°C for 1 h to allow the matrix solution to solidify. Late EPCs were harvested as described above with trypsin/EDTA, and then 1 × 10^4^ EPCs were placed in a matrix solution with EGM-2 MV medium and incubated at 37°C for 16 h. Tubule formation was inspected under an inverted light microscope (100×). Five representative fields were taken, and the average of the total area of complete tubes formed by cells was compared using computer software (Image-Pro Plus; Media Cybernetics, Finchampstead, United kingdom.).

### Isolation of cell fractions

Cells were harvested, sonicated for 10 s at output 4 with a sonicator (Ultrasonics, New York, NY, USA), and centrifuged at 8000 rpm for 15 min at 4°C. Pellets were collected as the nuclear fraction. The supernatant was centrifuged at 14,000 rpm at 4°C for 60 min to yield pellets (membrane fraction) and the supernatant (cytosolic fraction).

### Proliferation assay

The proliferation of late EPCs was determined by MTT assay (Sigma-Aldrich). The late EPCs were supplemented with MTT (0.5 mg/mL; Sigma) and were incubated for 4 h for the proliferation assay. Blue formazan was dissolved in dimethyl sulfoxide and measured at 550/650 nm.

### Measurement of intracellular ROS accumulation

The intracellular H_2_O_2_ levels were determined by measuring the fluorescence of dichlorofluorescein diacetate (DCFH-DA). The fluorescence for dichlorofluorescein (DCF) was detected at 495/529 nm. For the purpose of these experiments, late EPCs were washed with warm Hank’s buffered salt solution (HBSS) and incubated in HBSS or cell medium containing 10 mol/L DCFH-DA at 37°C for 45 min. Subsequently, HBSS or medium containing DCFH-DA was removed and replaced with fresh medium. Late EPCs were then incubated with various inhibitors and PMA. Cells were washed twice with PBS and lysed in a buffer (20% alcohol, 0.1% Tween 20, and 80% PBS), and the fluorescence intensity of the cells was measured using a microplate reader (TECAN Infinite 200 PRO; Tecan, Männedorf, Switzerland).

### Data analysis

Concentration-effect curves were fitted, and EC50 values were estimated using GraphPad Prism (GraphPad, San Diego, CA, USA). Data were expressed as the mean ± the standard error of the mean (SEM) and were analyzed by one-way analysis of variance followed by Tukey’s *post hoc* test. *P*<0.05 was considered significant.

## Results

### PMA enhanced the migration and tube formation of human late EPCs by activating MMP-9 and MMP-2 expressions

**Fig 1:**
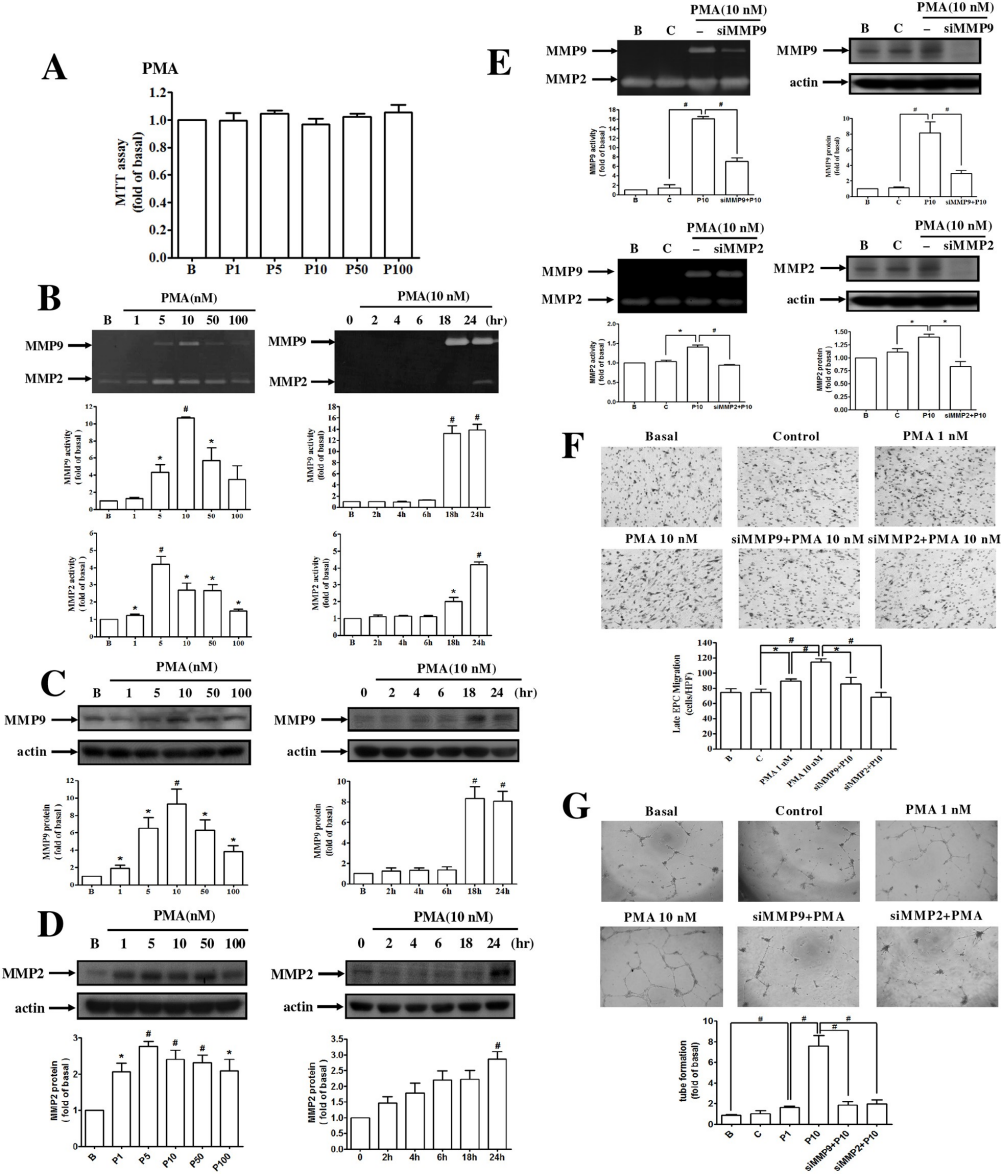
PMA enhanced the migration and tube formation of human late EPCs by activation of MMP-9 and MMP-2 expression. **(A)** Cells were incubated with different doses of PMA for 24 h, and cytotoxicity was measured by cell viability assay. **(B, C, D)** Cells were incubated with different doses of PMA for 24 h and 10 nM PMA for the indicated time intervals. The enzyme activities and protein levels of MMP-9 and MMP-2 were determined by **(B)** zymography and **(C, D)** Western blot analysis. **(E)** Cells were transfected with either control siRNA or MMP-9 siRNA and then incubated with PMA for 24 h. The levels of MMP-9 activity and protein were determined by zymography and Western blot. **(F, G)** Late EPCs were transfected with control, MMP-9, and MMP-2 siRNAs and then incubated with PMA for 24 h. The migration and tube formation were measured. Data are expressed as mean ± SEM of three independent experiments. Significant differences between the compared groups are indicated: *P < 0.05; ^#^*P* < 0.01.

The concentration of PMA used in this study did not alter the cell viability of late EPCs (Fig 1A). Treatment with PMA increased the activity and expression of MMP-2 and MMP-9 in EPCs compared with the control group (Fig 1B–1D) and MMP-9 activity was suppressed by siRNA transfection (Fig 1E). The migration and vascular formation caused by EPCs were significantly increased after PMA treatment. Suppression of MMP-2 and MMP-9 by SiRNA inhibited the migration and capillary vascular formation caused by the PMA-stimulated EPCs (Fig 1F–1G).

### PMA enhanced the migration and tube formation of human late EPCs by activation of PKC

**Fig 2:**
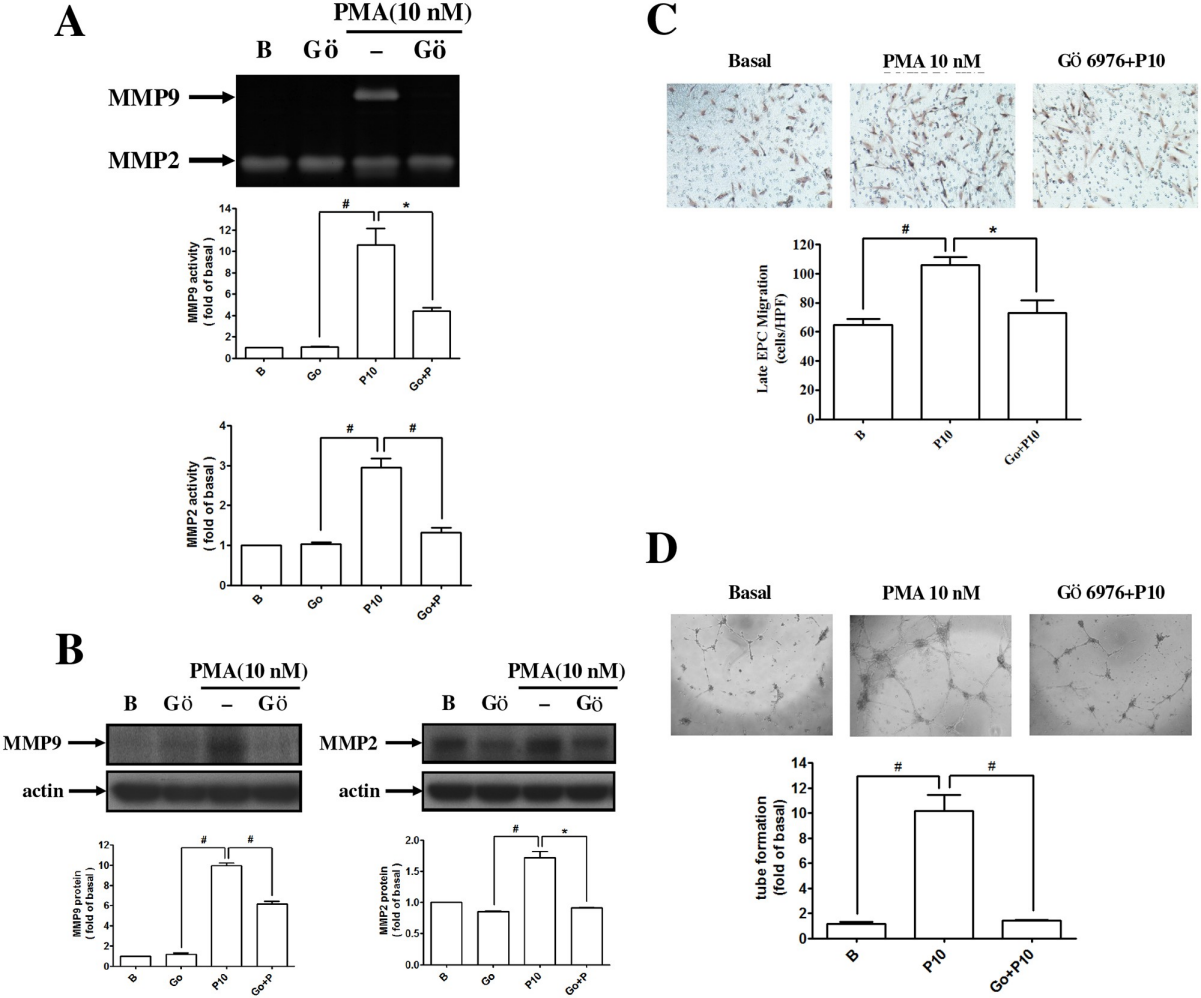
PMA enhanced the migration and tube formation of human late EPCs by activating PKCαpathways. **(A, B, C)** Cells were pretreated with 10 nM Gö6976 (a PKCα inhibitor) for 1 h and then treated with PMA for 24 h. The enzyme activities and protein levels of MMP-9 and MMP-2 were determined by **(A)** zymography and **(B)** Western blotting. **(C, D)** Late EPCs were pretreated with Gö6976 for 1 h and then were incubated with PMA for 24 h. The **(C)** migration and **(D)** tube formation were measured. Data are expressed as mean ± SEM of three independent experiments. Significant differences between the compared groups are indicated: *P < 0.05; ^#^*P* < 0.01.

The expression and activity of MMP-2 and MMP-9 from EPCs were increased after PMA treatment. A PKCα inhibitor significantly inhibited the activity and expression of MMPs caused by PMA-treated EPCs (Fig 2A and 2B). The migration and vascular formation of the PMA-treated EPCs were also suppressed by the PKCα inhibitor (Fig 2C and 2D).

### PMA enhanced MMPs expression, migration, and vascular formation of EPCs by redox-sensitive NF-κB pathways

**Fig 3:**
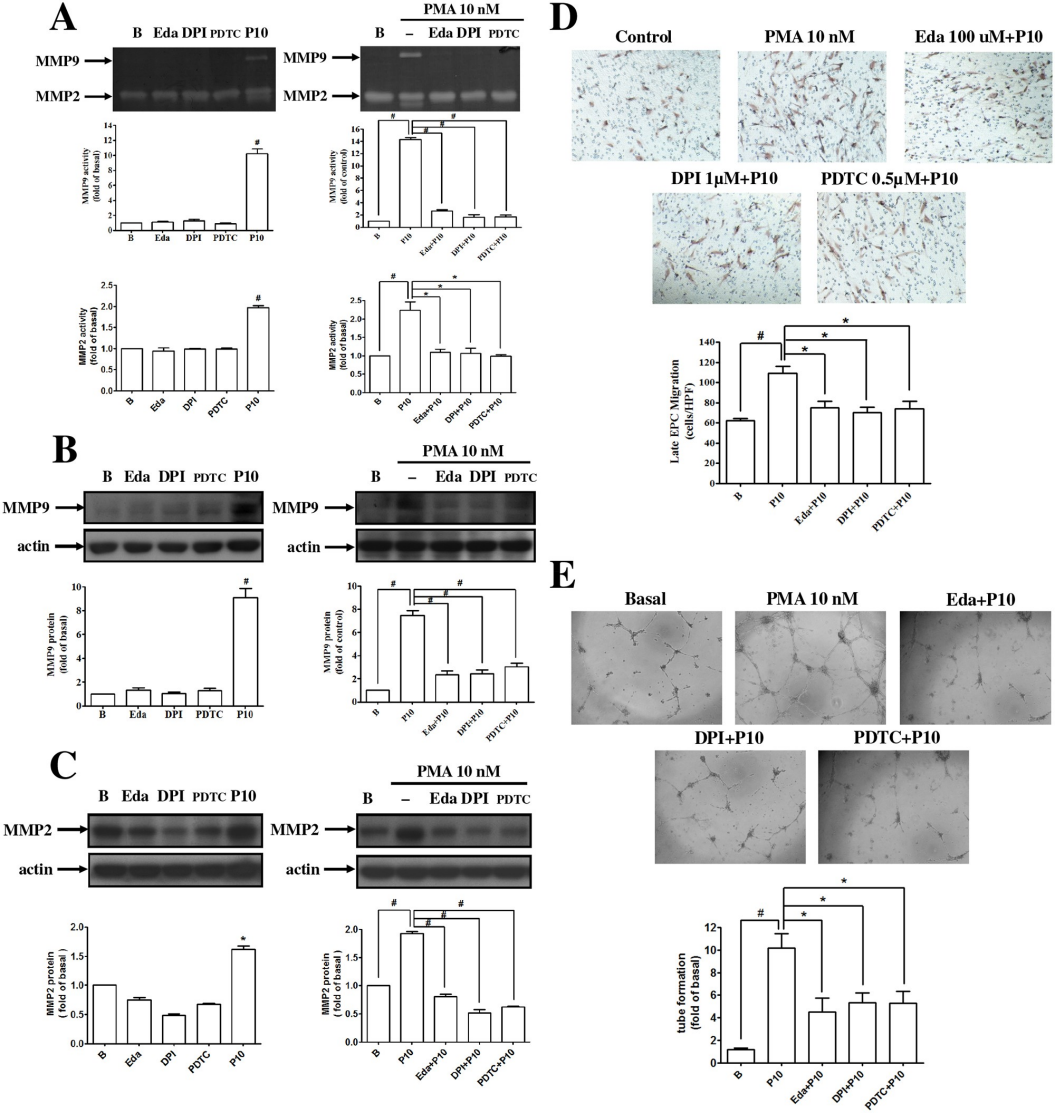
PMA induced MMP2 and MMP9 through p47phox/ROS in human late EPCs. **(A, B, C)** Cells were pretreated with 100 μM Edaravone (free radical scavenger), 5 μM DPI (a NADPH oxidase inhibitor), 0.5 μM PDTC (a NF-κB inhibitor) for 1 h, and then treated with PMA for 24 h. The activities and protein levels of MMP-9 and MMP-2 were determined by **(A)** zymography and **(B, C)** Western blotting. **(D, E)** Late EPCs were pretreated with Edaravone, DPI, and PDTC for 1 h and then incubated with PMA for 18 h. The migration and tube formation were measured. Data are expressed as mean ± SEM of three independent experiments. Significant differences between the compared groups are indicated: *P < 0.05; ^#^*P* < 0.01.

PMA enhanced the activity and expression of MMP-2 and MMP-9 by EPCs. After treatments with a free radical scavenger, an NADPH oxidase inhibitor, or an NF-κB inhibitor, the activity and expression of MMP-2 and MMP-9 were significantly reduced in PMA-treated EPCs, respectively (Fig 3A–3C). The angiogenic activities of the PMA-treated EPCs were significantly suppressed following treatment with the ROS inhibitors (Fig 3D and 3E).

### PMA enhanced MMPs expression, migration, and vascular formation of EPCs via the activation of NADPH oxidase

**Fig 4:**
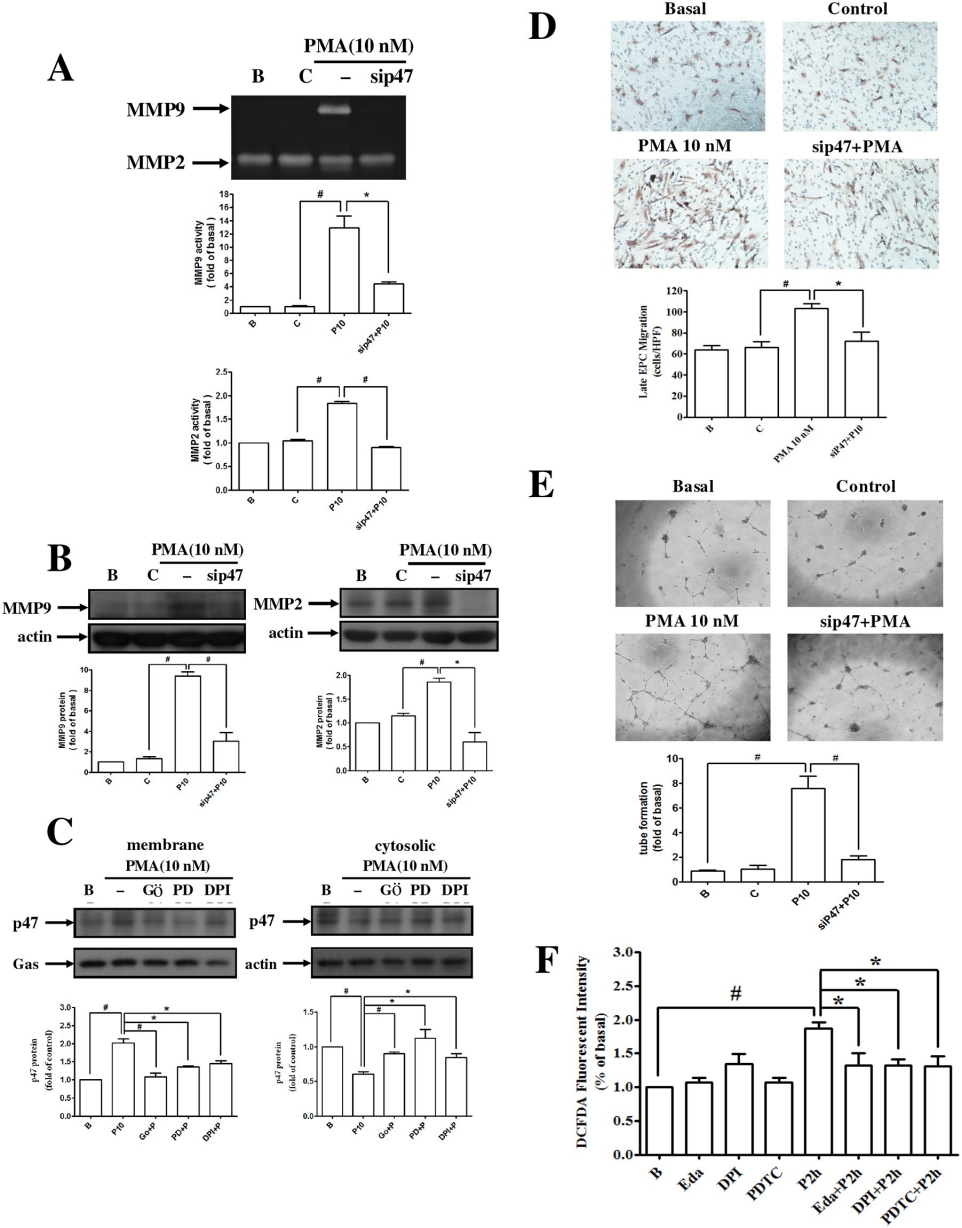
PMA enhanced MMP-2 and MMP-9 expression and migration and tube formation of late EPCs through activation of NADPH oxidase p47phox. **(A, B)** Cells were transfected with p47phox siRNA and then incubated with PMA for 24 h. The levels of MMP-9 activity and protein were determined by **(A)** zymography and **(B)** Western blot. **(C)** Late EPCs were pretreated with Gö6976 (a PKCα inhibitor), PDTC (a NF-κB inhibitor), and DPI (a NADPH oxidase inhibitor) for 1 h and then incubated with PMA for 30 min. Membrane and cytosolic extracts were prepared and subjected to Western blot using anti–p47phox antibodies. **(D, E)** Late EPCs were transfected with p47phox siRNA and then incubated with PMA for 18 h. The **(D)** migration and **(E)** tube formation were measured. **(F)** Cells were pretreated with Edaravone (a free radical scavenger), DPI, and PDTC for 1 h and then were treated with PMA for 2 h. DCF fluorescence intensities were measured using a flow cytometer. Data are expressed as mean ± SEM of three independent experiments. Significant differences between the compared groups are indicated: *P < 0.05; ^#^*P* < 0.01.

The activity and expression of MMP-2 and MMP-9 from PMA-treated EPCs were significantly reduced after the suppression of p47 by NADPH oxidase (Fig 4A and 4B). PMA increased the translocation of p47 caused by NADPH oxidase from the cytosol to the membrane of EPCs. PMA promoted the angiogenic activity of EPCs under ROS generation (Fig 4F). The migration and vascular formation of PMA-treated EPCs were inhibited by the suppression of p47 by NADPH oxidase (Fig 4D and 4E). These results demonstrated that NADPH oxidase -derived ROS mediated the migration and vascular formation of PMA-treated EPCs.

### PMA enhanced MMPs expression, migration, and vascular formation of EPCs by activating MAPK pathways

**Fig 5:**
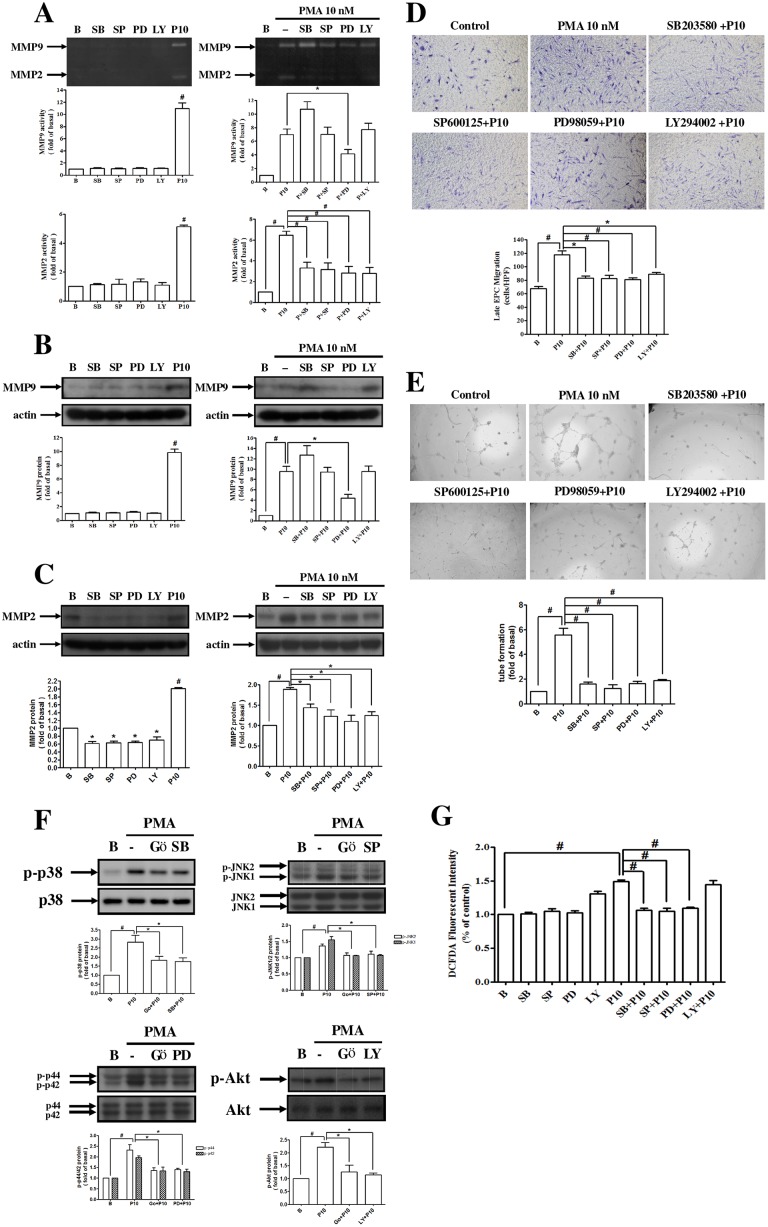
PMA enhanced MMP-2 and MMP-9 expression and migration and tube formation of EPCs via activation of MAPK pathways. **(A, B, C)** Cells were pretreated with 5 μM SB203580 (a p38 inhibitor), 5 μM SP600125 (a JNK inhibitor), 5 μM PD98059 (an Erk inhibitor), and 3 μM LY294002 (an Akt inhibitor) for 1 h, and then treated with PMA for 24 h. The activities and protein levels of MMP-9 and MMP-2 were determined by **(A)** zymography and **(B, C)** Western blot analysis. **(D, E)** Human (late outgrowth) EPCs were pretreated with SB203580, SP600125, PD98059, and LY294002 for 1 h and then incubated with PMA for 18 h. The **(D)** migration and **(E)** tube formation were measured. **(F)** Cells were pretreated with SB203580, SP600125, PD98059, and LY294002 for 1 h and then stimulated with PMA for 30 min. The cell lysates were subjected to Western blot using anti–phospho-p38, anti–phospho-SAPK/JNK, anti–phospho-Erk1/2, and anti–phospho-Akt antibodies. **(G)** Cells were pretreated with SB203580, SP600125, PD98059, and LY294002 for 1 h and then were treated with PMA for 2 h. DCF fluorescence intensities were measured with a flow cytometer. Data are expressed as mean ± SEM of three independent experiments. Significant differences between the compared groups are indicated: *P < 0.05; ^#^*P <* 0.01.

**Fig 6:**
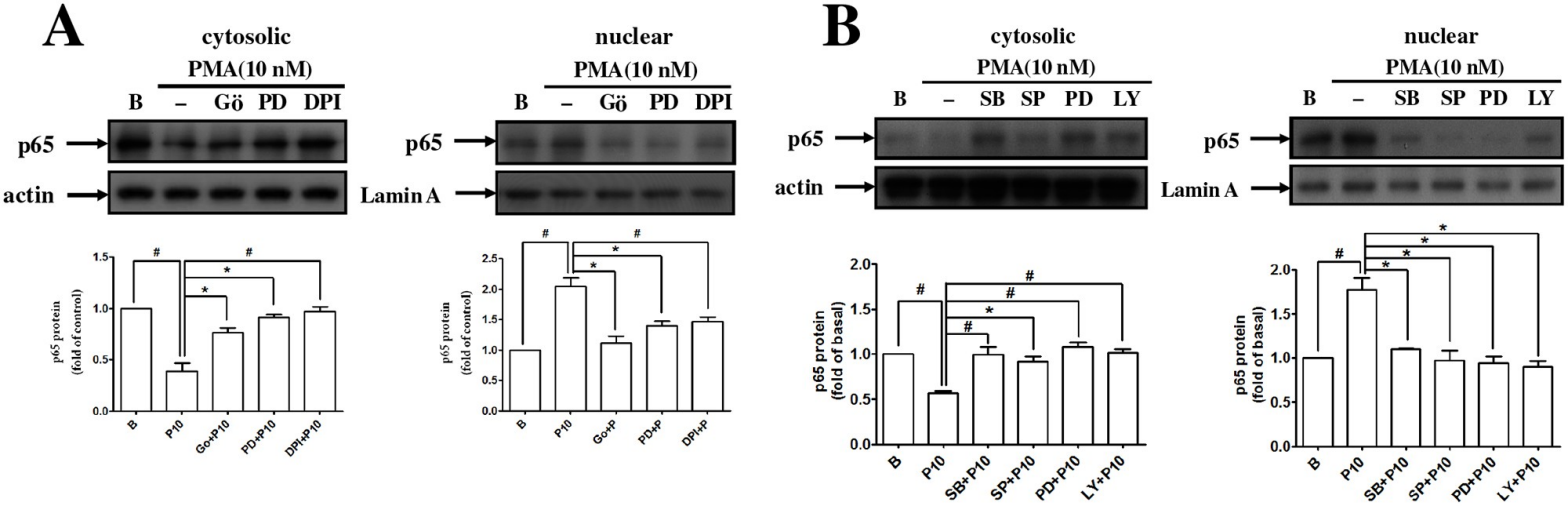
NF-κB activation was involved in PMA-induced MMP-2 and MMP-9 expression. **(A, B)** Human (late outgrowth) EPCs were pretreated with Gö6976 (a PKCα inhibitor), SB203580 (a p38 inhibitor), SP600125 (a JNK inhibitor), PD98059 (an Erk inhibitor), LY294002 (an Akt inhibitor), and DPI (an NADPH oxidase inhibitor) for 1 h and then were incubated with PMA for 30 min. Membrane and cytosolic extracts were prepared and subjected to Western blot using anti–NF-κB p65phox antibodies. Data are expressed as mean ± SEM of three independent experiments. Significant differences between the compared groups are indicated: *P < 0.05; ^#^*P* < 0.01.

The activity and expression of MMP-9 caused by PMA-treated EPCs were significantly inhibited by treatment with an Erk inhibitor. However, the activity and expression of MMP-2 from PMA-treated EPCs were suppressed by p38, JNK, Erk, and Akt inhibitors (Fig 5A–5C). The migration and vascular formation of the PMA-stimulated EPCs were inhibited by these MAPK inhibitors (Fig 5D and 5E). We examined the effect of these MAPK inhibitors on the NOX-mediated ROS, and found that the p38, JNK, and Erk inhibitors were involved, but the Akt inhibitor was not (Fig 5F and 5G). The translocation of p65 in the PMA-treated EPCs from the cytosol to the nuclear region was also evaluated with various MAPK inhibitors (Fig 6).

### Transplantation of PMA-treated late EPCs promoted angiogenesis in a mouse hindlimb ischemia model

**Fig 7:**
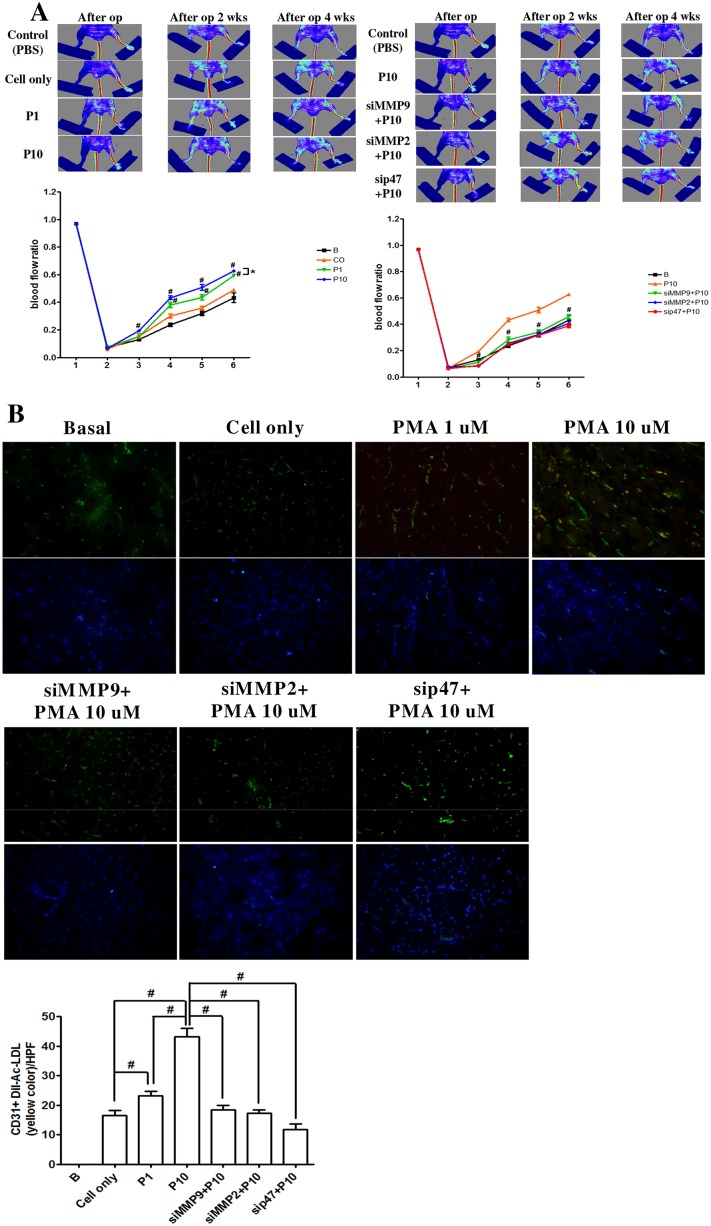
PMA-stimulated late EPCs could improve angiogenesis in a hindlimb ischemia model. **(A)** Representative images of blood flow were measured by a laser Doppler perfusion image (LDPI) analyzer. Quantitative analysis of the blood flow recovery was measured by LDPI. The LDPI index was calculated as the ratio of ischemic to non-ischemic hindlimb blood flow (n = 6 per group). **(B)** Representative immunohistostaining of hindlimb sections 4 weeks after Dil-labeled cells (red fluorescence) and stained CD31 (green fluorescence) antibodies in hindlimb sections. Dil-labeled cells (red fluorescence) late EPCs after cell implantation and CD31 (green fluorescence) staining of ischemic hindlimb muscles were quantified (yellow color). The nuclei were stained with DAPI (blue fluorescence) in the hindlimb muscle (n = 6 per group). Data are expressed as mean ± SEM of three independent experiments. Significant differences between the compared groups are indicated: *P < 0.05; ^#^*P* < 0.01.

To evaluate the effect of PMA and MMPs in angiogenesis, we used a nude mouse model of ischemia-induced angiogenesis. Compared with the control mice, the PMA-treated mice had better blood flow recovery after surgery (Fig 7A); however, the administration of MMP-2 and MMP-9 siRNA significantly impaired flow recovery in the PMA-stimulated mice. Anti–CD31 immunostaining showed increased capillary density in the PMA-treated mice compared with the control mice, but treatment with MMPs siRNA significantly decreased the capillary density in the mice muscles (Fig 7B). These results suggested that PMA induced ischemic neovascularization via up-regulation of the expressions of MMP-2 and MMP-9.

## Discussion

**Fig 8:**
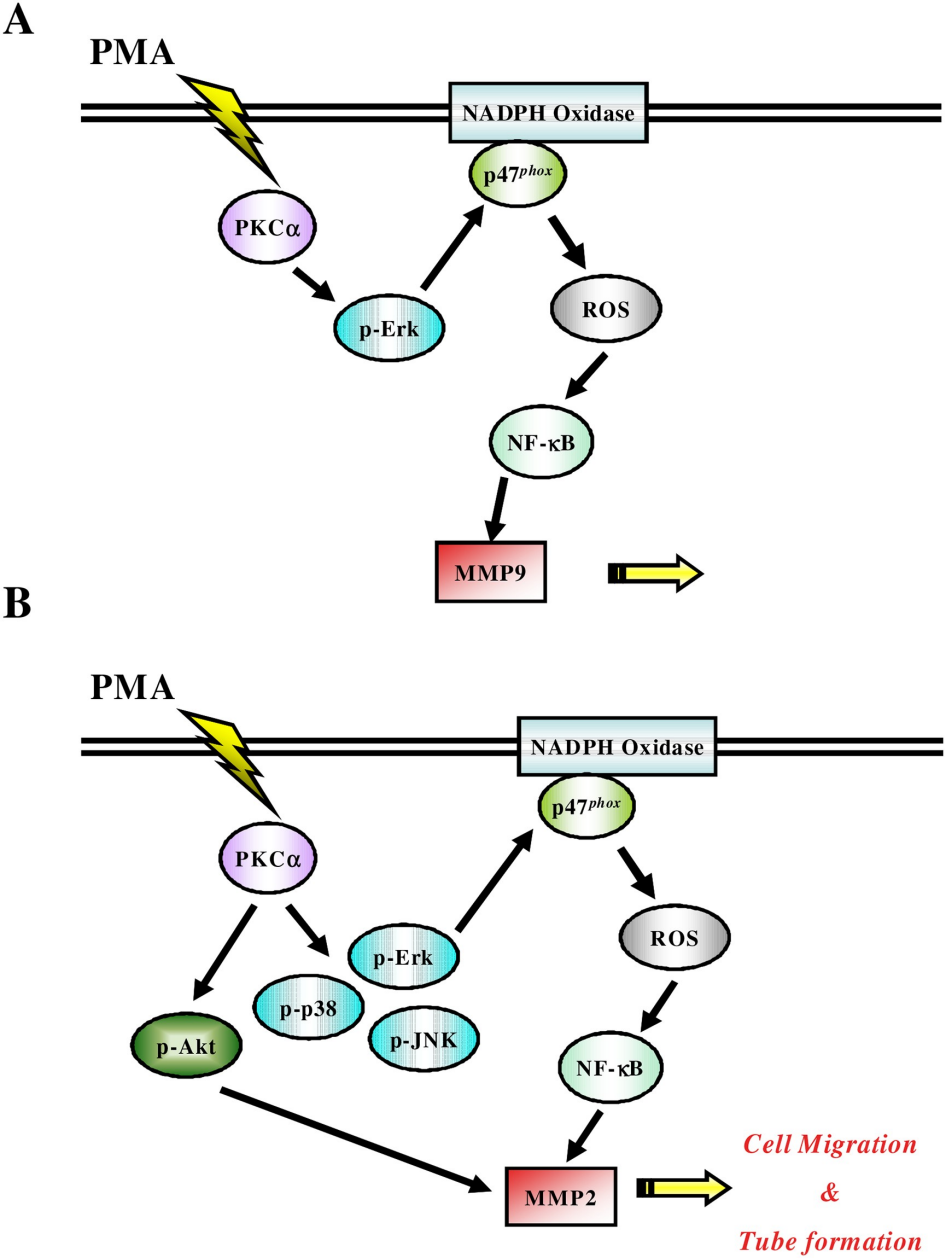
The proposed signaling pathways involved in PMA-induced MMP-2/-9-dependent cell migration and tube formation of human late EPCs. **(A)** PMA activates the PKCα/Erk1/Erk2/NADPH oxidase pathway to enhance ROS generation, which in turn initiates the activation of NF-κB and ultimately induces MMP-9 expression in late EPCs. **(B)** PMA activates the PKCα/MAPKs/NADPH oxidase/ROS/NF-κB or PKCα/Akt pathways to induce MMP-2 expression, which may further enhance cell migration and tube formation of EPCs.

The major findings of the current study are (1) the expression and activities of MMP-2 and MMP-9 were increased in PMA-stimulated EPCs; (2) PMA can enhanced the migration and tube formation of EPCs caused by the expression of MMP-2 and MMP-9; (3) a PKCα inhibitor suppressed the effect of PMA; (4) the PMA-induced angiogenesis function of EPCs caused by the expressions of MMPs could be inhibited by the reducing oxidative stress via NADPH oxidase and MAPK pathways; and (5) transplantation of PMA-treated human EPCs promoted angiogenesis in a mouse hindlimb ischemia model. Taken together, our findings demonstrate the novel redox-related mechanisms (PKCα—NADPH oxidase–NF-κB- MMP-9) by which PMA could enhanced EPC function via activating MMP-9 and also the complex mechanisms (PKCα—NADPH oxidase–NF-κB–MMP-2 and PKCα –Akt–MMP-2) by which PMA could enhance EPC function via activating MMP-2 (Fig 8). These findings also suggest a potential therapeutic approach to enhance human EPC function for angiogenesis.

The findings of the current study are in line with our previous findings that MMP-9 can enhance the angiogenic activities of EPCs, including migration, proliferation, and release of VEGF [13]. Ischemia-induced neovascularization can be impaired through the dysfunction of bone marrow–derived EPCs due to a deficient expression of MMP-9 [3, 4,14]. On the other hand, Cheng et al. also showed that impairment of ischemia-induced neovascularization in MMP-2 knockout mice could be improved by transplantation of bone marrow–derived monocytes from MMP-2(+) mice [15]. In the present study, the mobilization and vascular formation of EPCs were impaired after the suppression of MMP-2 and MMP-9, which were then recovered after PMA treatment, suggesting the potential use of PMA to enhance human EPC function by up-regulating MMP-2 and -9 expressions. While different vascular strategies may be considered in differential clinical conditions from the recovery of limb ischemia to the suppression of cancer growth, it would be interesting to investigate whether the expressions of MMP-2 and MMP-9 expressions in EPCs could be optimized for either pro-angiogenesis or anti-angiogenesis depending on the clinical indications.

It has been shown that ROS are important mediators and modulate various signaling pathways in angiogenesis, including up-regulation of VEGF expression [16], HIF [17], and urotensin-II [18]. Low levels of NADPH oxidase-related oxidative stress may promote EPC migration and differentiation to induce neovascularization [19]. In the current study, PMA induced MMP-2 and MMP-9 expression in response to oxidative stress to promote angiogenesis. NADPH oxidases are known to be a major source of ROS in endothelial cells and progenitor cells, and membrane translocation of p47phox has been shown to play an important role in the activation of NADPH oxidase [20]. Our findings indicate that PMA may augment ROS-dependent MMP-2 and MMP-9 expression via different intracellular pathways in EPCs. With regards to the expression of MMP-9 in EPCs, PMA induced intracellular ROS production of NADPH oxidase by activating PKCα and the Erk signaling pathways. On the other hand, with regards to the expression of MMP-2 in EPCs, PMA induced ROS production of NADPH oxidase via activation of complex signaling pathways involving PKCα, p38, Akt, Erk, and JNK. Nevertheless, the redox-dependent NF-κB activation is commonly required for PMA-induced MMP-2 and MMP-9 expressions in the EPCs (Fig 8, S1 File).

It has been suggested that PKCs may increase atherosclerotic changes in diabetes [21]. However, the effect of PKCs on angiogenesis is controversial. Cortif et al. showed that PKC promotes the angiogenic activity of endothelial cells [22], and Moncada de la Rosa et al demonstrated the inhibitory effect of PKCs in platelet-related angiogenesis [23]. Moriya et al. also found that up-regulation of PKCs impaired the platelet-induced angiogenesis of endothelial cells in hyperglycemia [12]. In addition, Montesano and Orci reported that PMA may induce angiogenesis in vitro from large-vessel endothelial cells [24]. Furthermore, they showed that PMA led to activation of the PKC isoforms α, δ, and ε in human umbilical vein endothelial cells. Knockdown of PKCα has also been shown to diminish PMA-induced VEGF expression and angiogenesis [10] and PKCα has been shown to stimulate nitric oxide production in the regulation of blood flow [25].

Thus, PMA-induced PKCα activation may promote the angiogenic activity of human endothelial cells via induction of VEGF [10]. However, in the present study, the expression of PKCα was significantly up-regulated in EPCs by PMA treatment, which then increased MMP-2 and MMP-9 expressions for neovascularization, suggesting the novel mechanisms of angiogenic activity in human EPCs. PKC is a family of multifunctional isoenzymes involved in inflammation, platelet function [26] and cardiac function [27]. Accordingly, the mechanistic effects of PKC on angiogenesis seem complicated. Although PMA also activated PKCβ, the angiogenic effect of PKCα was stronger than PKCβ in the current study. PKCα was more important than PKCβ in angiogenesis in the present study (S1 Fig, S2 File). However, Shih YH et al found that a PKCα-NF-κB-dependent cascade was involved in the signaling leading to PMA-induced MMP-9 expression in the lung epithelial cells [28]. Scoditti E et al presented that hydroxytyrosol reduced MMP-9 induction in activated human monocytes via PKCα and PKCβ1 inhibition by PMA stimulation [29]. Different isoenzymes of PKC may play different roles in angiogenesis in diverse stem cell types under various conditions. Future studies are indicated to clarify the potential impacts of PKC isoenzymes in cardiovascular disease [30].

## Conclusions

In conclusion, both redox-related MMP-2 and MMP-9 activation are critical to the function of EPCs for in vivo ischemia-induced angiogenesis. Treatment with PMA enhanced the function of EPCs for angiogenesis via the activation of MMP-9 by PKCα—NADPH oxidase–NF-κB pathways, and via the activation of MMP-2 by PKCα—NADPH oxidase–NF-κB and PKCα –Akt pathways. The complex mechanisms of PMA-activated EPCs for neovascularization may provide some novel insights into different therapeutic strategies for either pro- or anti-angiogenesis in various clinical conditions.

## Supporting information

S1 Fig PMA enhanced the migration and tube formation of human late EPCs by activating PKC α and β pathways. **(A, B, C, D)** Cells were pretreated with the PKC α and β inhibitors for 1 h and then treated with PMA for 24 h. The enzyme activities and protein levels of MMP-9 and MMP-2 were determined by **(A)** zymography and **(B)** Western blotting. The **(C)** migration and **(D)** tube formation were measured. Data are expressed as mean ± SEM of three independent experiments. Significant differences between the compared groups are indicated: *P < 0.05; ^#^*P* < 0.01.
(PDF)

S1 File The original data of western blotting. (PPTX)

S2 File The effect of PMA on angiogenic function of late EPCs by activating PKC α and β pathways. (PPTX)

## Abbreviations

Akt: protein kinase B
DCF: dichlorofluorscein
DCFH-DA: dichlorofluorscein diacetate
DPI: NADPH oxidase inhibitor
eNOS: endothelial nitric oxide synthase
EPC: endothelial progenitor cell
Erk: extracellular signal–regulated kinase
FITC: fluorescein isothiocyanate
HBSS: Hank’s buffered salt solution
HIF-1: hypoxia inducible factor-1
JNK: c-Jun N-terminal kinase
LDPI: laser Doppler perfusion image analyzer
MAPK: mitogen-activated protein kinase
MMP: matrix metalloproteinase
MNC: monocyte
NADPH: nicotinamide adenine dinucleotide phosphate
NF-κB: nuclear factor κB
NOX: NADPH oxidase
PBS: phosphate-buffered saline
PDTC: NF-κB inhibitor
PKCα: protein kinase C α (EPCs)
PMA: phorbol-12-myristate-13-acetate
ROS: reactive oxygen species
SDS-PAGE: sodium dodecyl sulfate–polyacrylamide gel electrophoresis
SEM: standard error of the mean
siRNA: small interfering RNA
VEGF: vascular endothelial growth factor
