# Supplementary figures and images for: Phorbol ester-induced angiogenesis of endothelial progenitor cells: The role of NADPH oxidase-mediated, redox-related matrix metalloproteinase pathways
Source: PLoS One. 2019 Jan 15;14(1):e0209426. doi: 10.1371/journal.pone.0209426 (PMC6333344; doi:10.1371/journal.pone.0209426)

Supplement

A

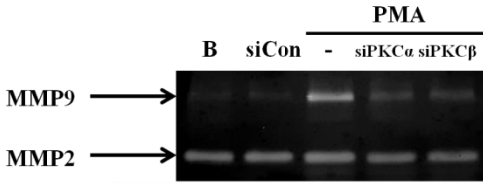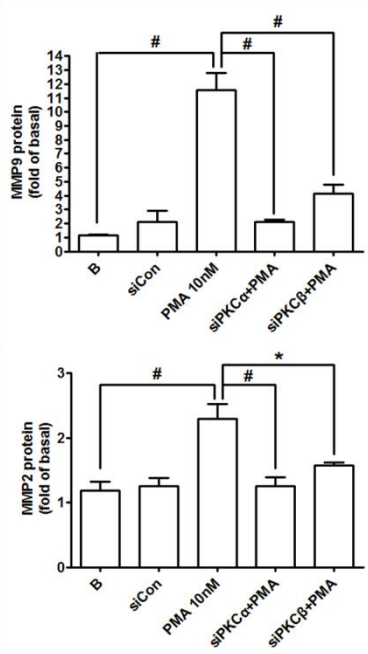

B

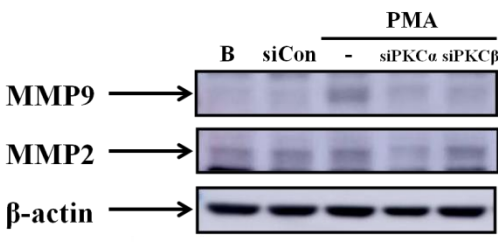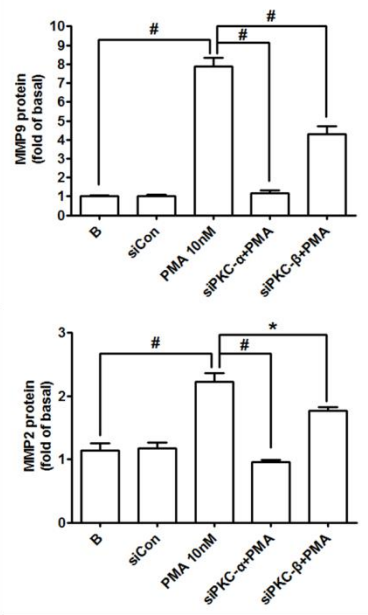

C

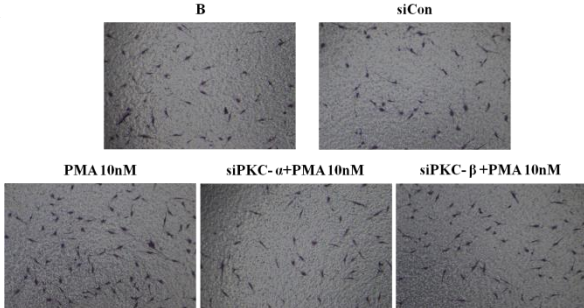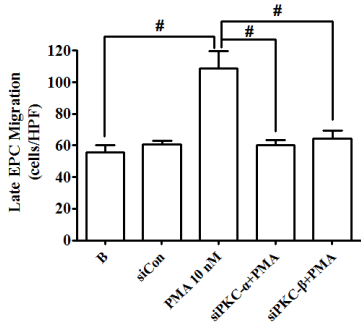

D

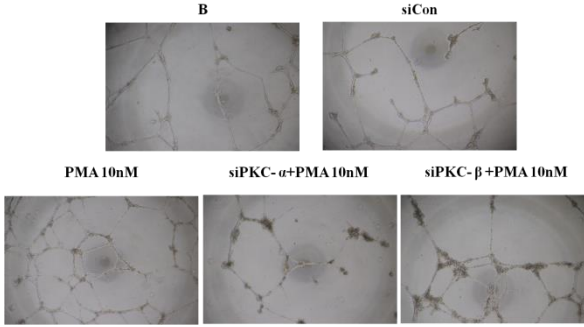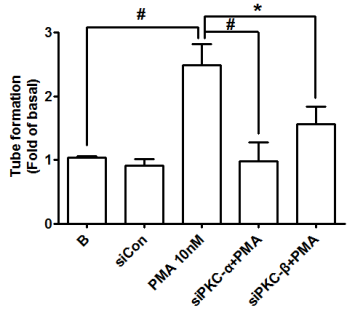

Supplement: S1 Fig — (A, B, C, D) Cells were pretreated with the PKC α and β inhibitors for 1 h and then treated with PMA for 24 h. The enzyme activities and protein levels of MMP-9 and MMP-2 were determined by (A) zymography and (B) Western blotting. The (C) migration and (D) tube formation were measured. Data are expressed as mean ± SEM of three independent experiments. Significant differences between the compared groups are indicated: *P < 0.05; #P < 0.01. (PDF) [file pone.0209426.s001.pdf]

## Slide 1
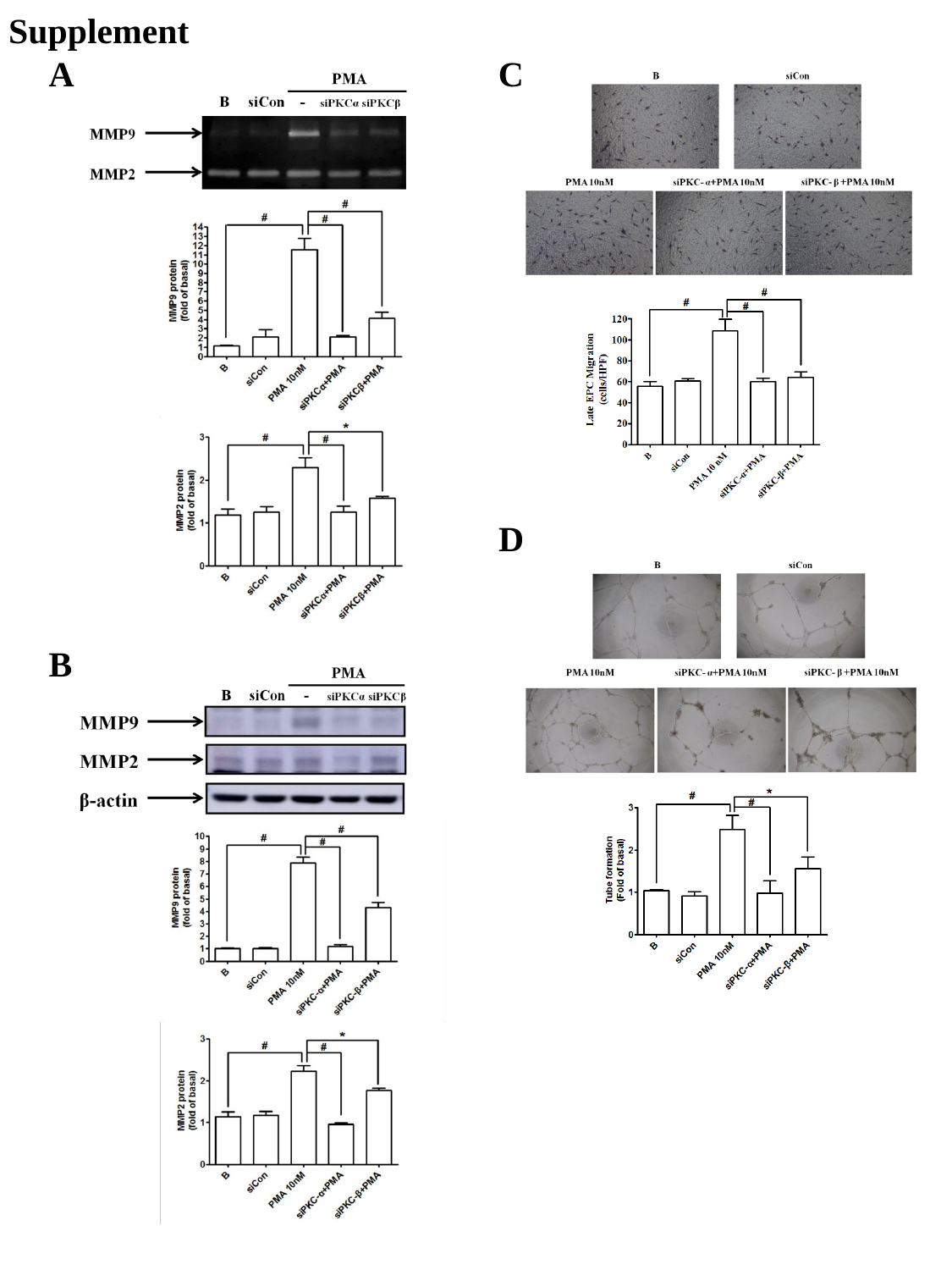

Supplement
A
C
D
B

Supplement: S2 File — (PPTX) [file pone.0209426.s003.pptx]
