# Supplementary material for: Phorbol ester-induced angiogenesis of endothelial progenitor cells: The role of NADPH oxidase-mediated, redox-related matrix metalloproteinase pathways
Source: PLoS One. 2019 Jan 15;14(1):e0209426. doi: 10.1371/journal.pone.0209426 (PMC6333344; doi:10.1371/journal.pone.0209426)
Supplement: S1 File — (PPTX) [file pone.0209426.s002.pptx]

## Slide 1
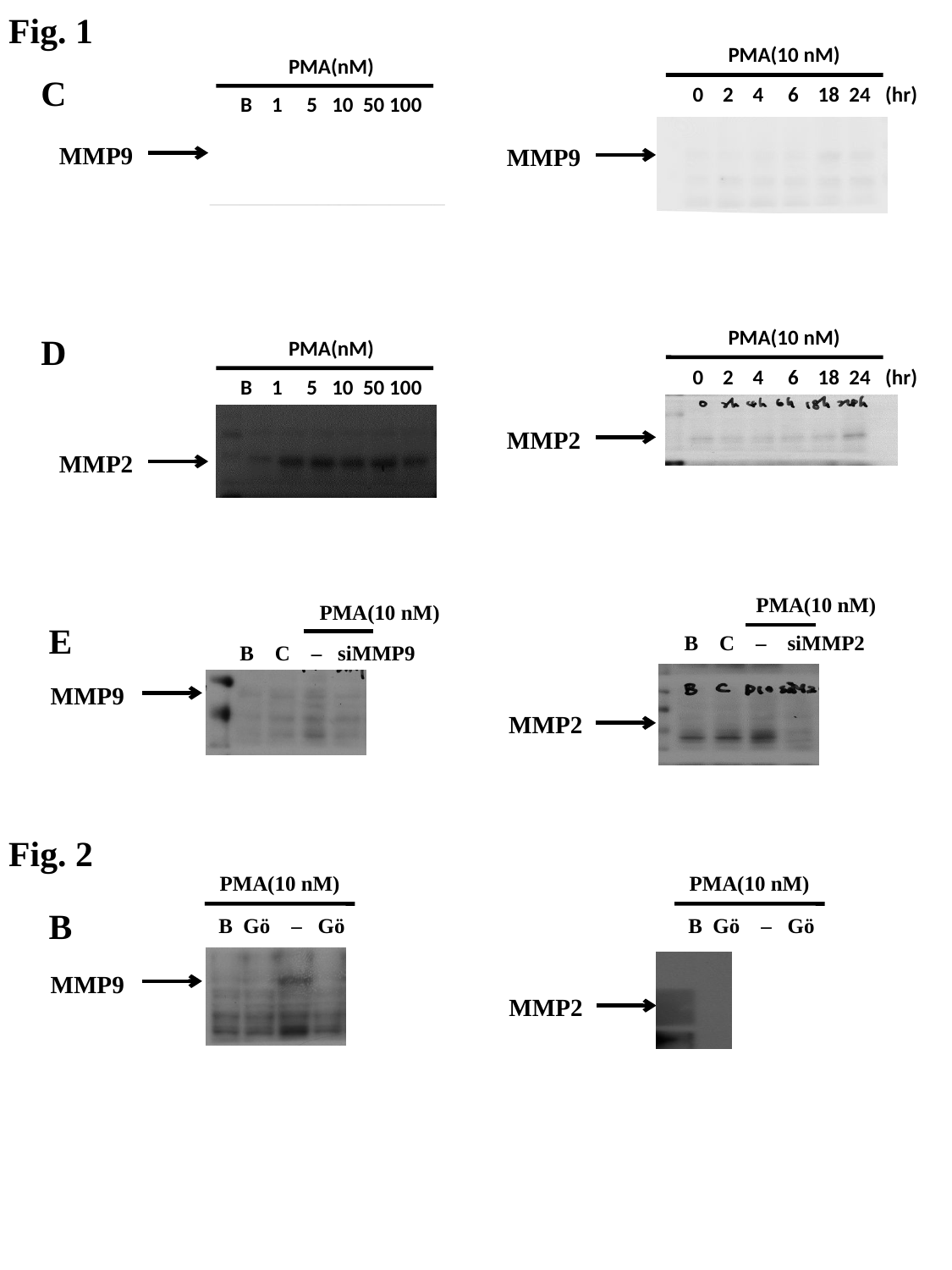

Fig. 1
PMA(10 nM)
PMA(nM)
C
 0 2 4 6 18 24 (hr)
 B 1 5 10 50 100
MMP9
MMP9
PMA(10 nM)
D
PMA(nM)
 0 2 4 6 18 24 (hr)
 B 1 5 10 50 100
MMP2
MMP2
PMA(10 nM)
PMA(10 nM)
E
 B C – siMMP2
 B C – siMMP9
MMP9
MMP2
Fig. 2
PMA(10 nM)
PMA(10 nM)
B
B Gö – Gö
B Gö – Gö
MMP9
MMP2

## Slide 2
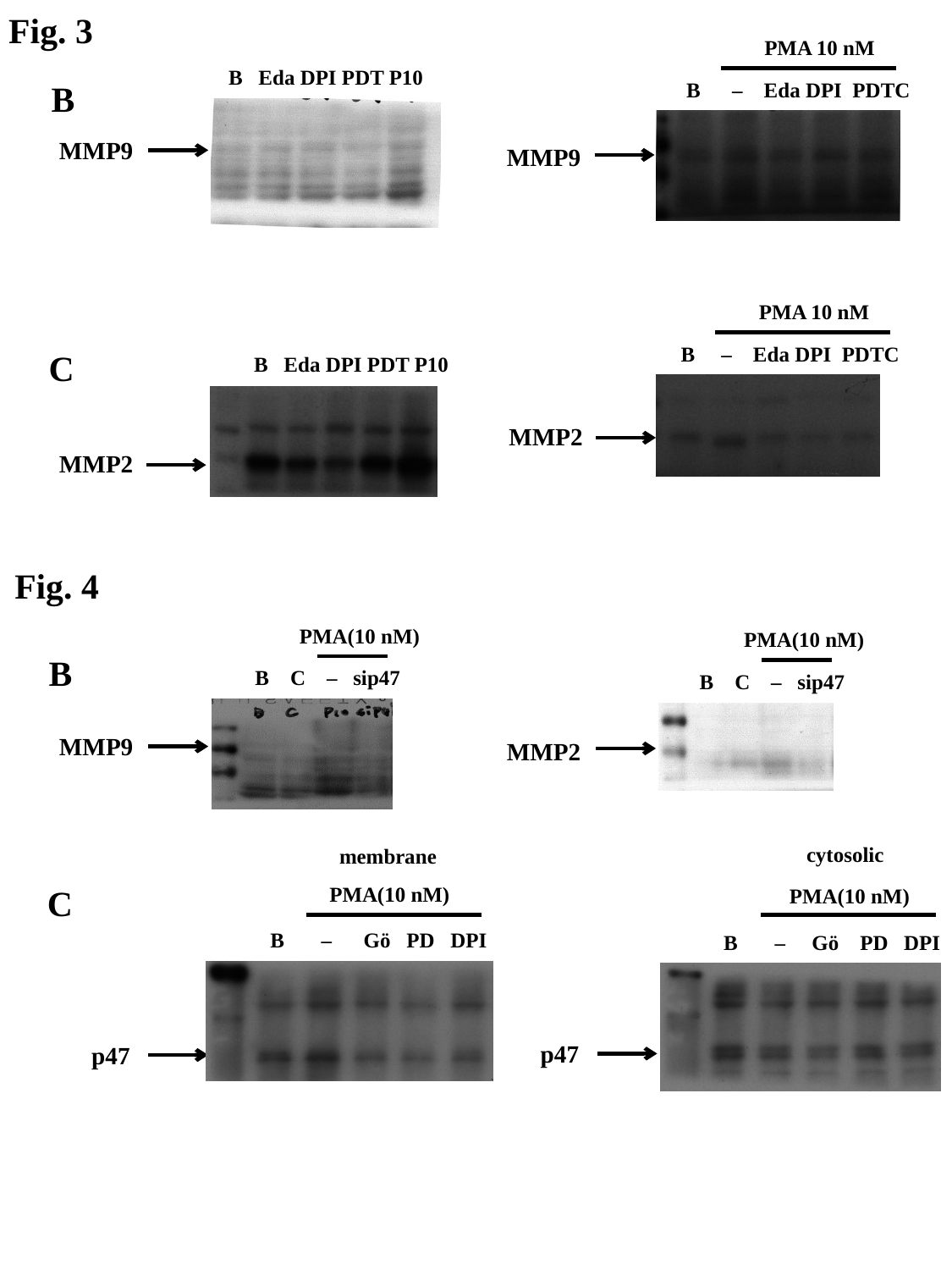

Fig. 3
PMA 10 nM
 B Eda DPI PDT P10
 B – Eda DPI PDTC
B
MMP9
MMP9
PMA 10 nM
 B – Eda DPI PDTC
C
 B Eda DPI PDT P10
MMP2
MMP2
Fig. 4
PMA(10 nM)
PMA(10 nM)
B
 B C – sip47
 B C – sip47
MMP9
MMP2
cytosolic
membrane
C
PMA(10 nM)
PMA(10 nM)
B – Gö PD DPI
B – Gö PD DPI
p47
p47

## Slide 3
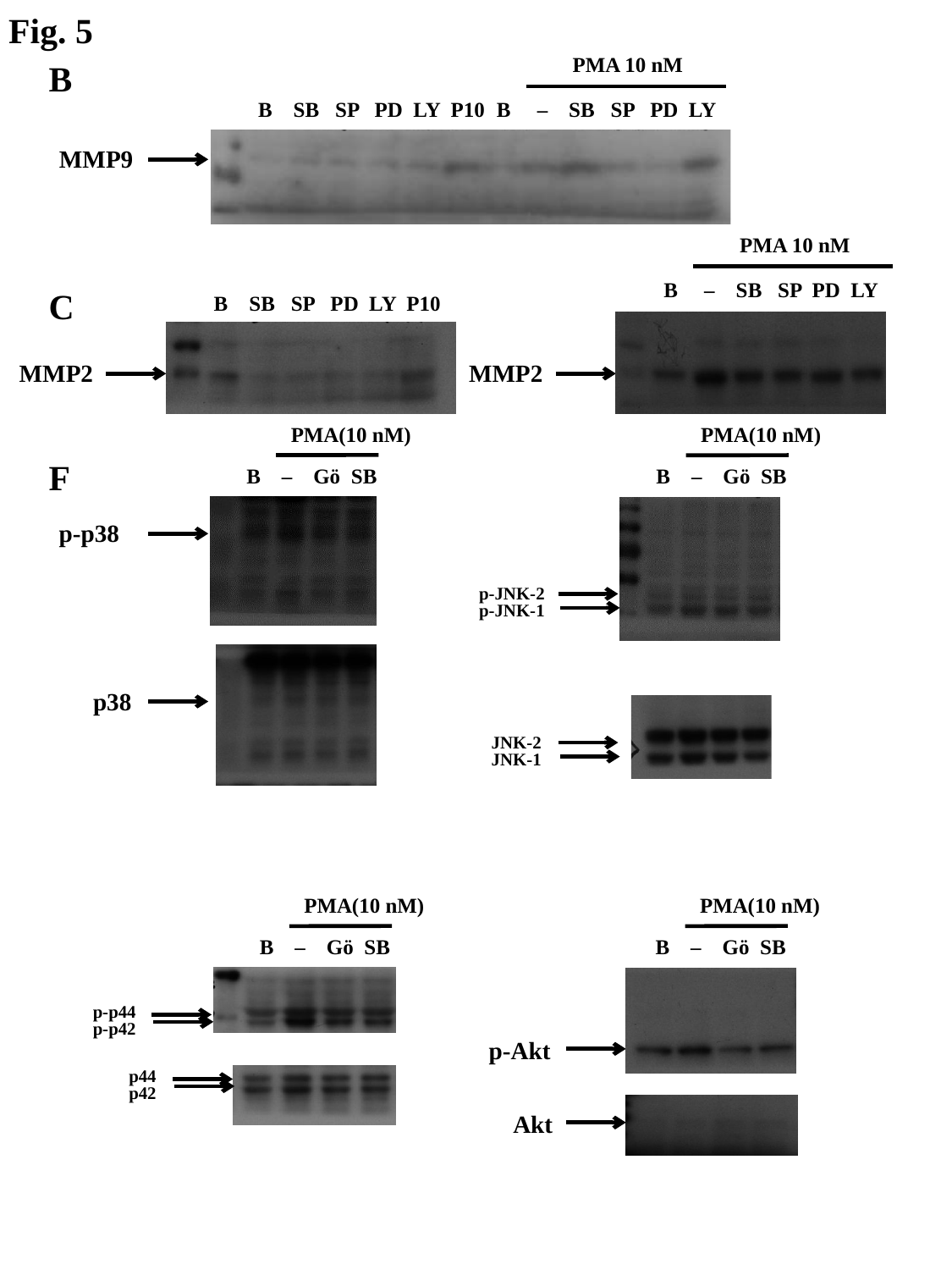

Fig. 5
PMA 10 nM
B
 B SB SP PD LY P10
 B – SB SP PD LY
MMP9
PMA 10 nM
 B – SB SP PD LY
C
 B SB SP PD LY P10
MMP2
MMP2
PMA(10 nM)
PMA(10 nM)
F
 B – Gö SB
 B – Gö SB
p-p38
p-JNK-2
p-JNK-1
p38
JNK-2
JNK-1
PMA(10 nM)
PMA(10 nM)
 B – Gö SB
 B – Gö SB
p-p44
p-p42
p-Akt
p44
p42
Akt

## Slide 4
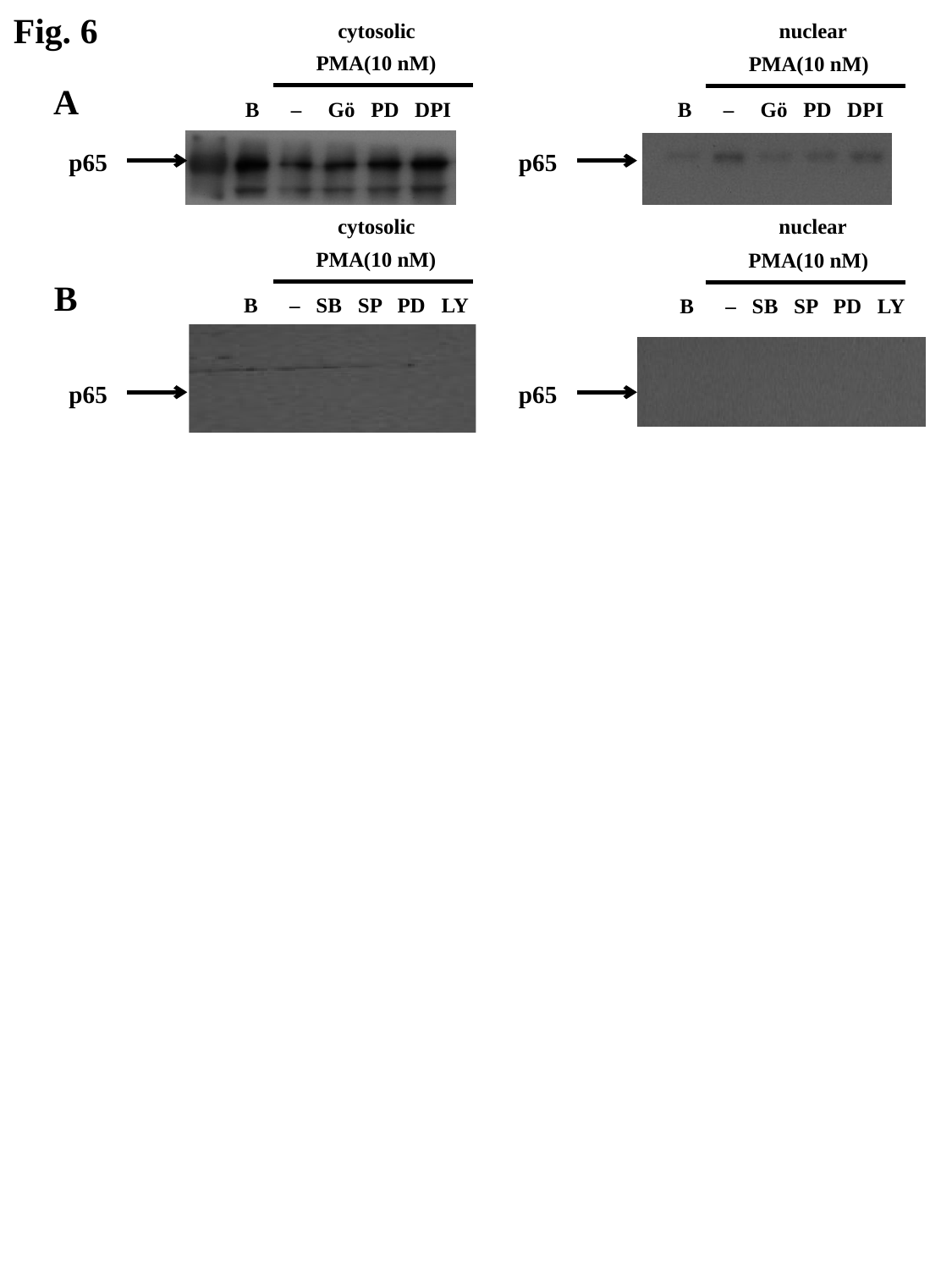

Fig. 6
cytosolic
nuclear
PMA(10 nM)
PMA(10 nM)
A
B – Gö PD DPI
B – Gö PD DPI
p65
p65
cytosolic
nuclear
PMA(10 nM)
PMA(10 nM)
B
B – SB SP PD LY
B – SB SP PD LY
p65
p65
